# Quality Evaluation of Biscuits Supplemented with Alfalfa Seed Flour

**DOI:** 10.3390/foods5040068

**Published:** 2016-10-31

**Authors:** Fahim Ullah, Sajjad Ahmad, Said Wahab, Alam Zeb, Mansoor Khan Khattak, Saleem Khan, Min Kang

**Affiliations:** 1 College of Engineering, Nanjing Agriculture University, Nanjing, 210031, China; fahimullah320@yahoo.com; 2 Department of Food Science and Technology, The University of Agriculture, Peshawar, Khyber 25000, Pakhtunkhwa, Pakistan; auisj95@gmail.com (S.A.); drsaidwahab40@yahoo.com (S.W.); drzebalam@hotmail.com (A.Z.); 3 Department of Agricultural Mechanization, The University of Agriculture, Peshawar, Khyber 25000, Pakhtunkhwa, Pakistan; mansoorkhankhattak@yahoo.com; 4 Department of Human Nutrition, The University of Agriculture, Peshawar, Khyber 25000, Pakhtunkhwa, Pakistan; saleem_khan288@yahoo.com; 5 Guanyun Research Institute for Modern Agricultural Equipment, Nanjing Agricultural University, Guanyun 222200, China

**Keywords:** alfalfa seed flour, supplemented biscuits, quality evaluation

## Abstract

The effect of alfalfa seed flour supplementation on the quality characteristics of refined wheat flour-based biscuits was studied. The proximate composition of refined wheat flour and alfalfa seed flour was determined. Refined wheat flour contained 12.43% moisture, 11.52% crude protein, 1.61% crude fat, 0.71% crude fiber, 1.43% ash and 70.83% nitrogen free extract, while alfalfa seed flour contained 5.79%, 29.49%, 12.71%, 5.53%, 4.80% and 41.73% moisture, crude protein, crude fat, crude fiber, ash and nitrogen free extract correspondingly. Alfalfa seed flour at 5%, 10%, 15% and 20% supplementation levels was incorporated in refined wheat flour to produce composite flour. The biscuits prepared were subjected to quality evaluation. Physical analysis of biscuits disclosed that supplementation of alfalfa seed flour decreased the width from 47.25 to 42 mm and the spread factor from 62.7 to 53.12, while it increased the thickness from 7.53 to 8.10 mm. Supplementation of refined wheat flour–based biscuits with alfalfa seed flour at different inclusion levels significantly (*p* < 0.05) increased the crude protein content from 10.19% to 15.30%, the crude fiber content from 0.73% to 1.62%, the crude fat content from 17.46% to 21.59% and the ash content from 1.37% to 1.92%, whereas it decreased the moisture content from 3.57% to 3.26% and the nitrogen free extract from 66.90% to 59.32%. The effect of supplementation on the mineral contents of biscuits was also significant (*p* < 0.05). Potassium, magnesium, calcium, iron and zinc contents increased from 105.30, 14.65, 43.91, 3.74 and 0.94 to 145.00, 26.64, 79.60, 7.93 and 1.60 mg/100 g, respectively. Sensory evaluation revealed that the quality score of biscuits in terms of color, taste, texture and overall acceptability decreased with increased supplementation. The present research work confirmed that a maximum of 10% alfalfa seed flour supplementation in refined wheat flour could produce acceptable biscuits with an appropriate nutritional profile.

## 1. Introduction

The word biscuit is derived from the Latin “bis coctus”, which means twice cooked. They are first positioned in a hot oven to bake and then transferred to dry out in a cool oven [1]. Biscuits and other baked products have been made and consumed by humans for centuries [2]. They are eaten before and after meals throughout the world as they are accepted and consumed by all sorts of peoples [3]. They are served in a semiformal situation with tea or coffee and are used as weaning food for infants. The per capita consumption of biscuits is growing progressively [4]. They command broad popularity in rural as well as urban vicinities between all the age groups [5].

A number of causes for such broad fame of biscuits are low price among other baked foods and straightforward accessibility [6]. Biscuits have low moisture content, are relatively free from microbial spoilage and have a long shelf life [7]. They provide a substantial source of energy and are eaten extensively throughout the world [8,9]. Mishra et al. [7] reported that in a short time the qualities of biscuits formulate enormous range production and its distribution.

Though the consumption of biscuits is very popular, the inferior quality protein of wheat flour has been an important concern in its utilization [10]. Its protein is deficient in essential amino acids such as lysine, threonine and tryptophan [11,12,13]. Refined wheat flour used for the preparation of biscuits is deficient in several nutrients, including dietary fiber, mineral elements as well as a few vitamins [14]. The deficiency of protein can be countered through protein-rich non-wheat flour substitution [6]. Mixing cereals with inferior nutritional value with legumes with superior nutritional value could help to solve the deficiency problems of cereals [15].

Legumes such as beans, peas and alfalfa are essential sources of dietetic protein for a large area of the world’s inhabitants [16,17]. They are extensively recognized as significantly consistent for the supplementation of cereal-based conventional bakery products such as biscuits, cakes and bread [18]. Adeyemi et al. [19] reported that cereal-based bakery products such as biscuits, cakes and breads, etc., are supplemented with non-wheat protein-rich flour such as legumes to improve their nutritional quality. Alfalfa (*Medicago sativa* L.) is an ancient crop. It belongs to the pea family Fabaceae [20]. The charred seeds have originated from archeological locations dating back 8000 years in Iran, while the charred seeds from small-seeded legumes and grasses gathered by humans date back 12,000 years in present-day Syria [21].

Alfalfa seeds are a rich source of protein and other nutrients on average; they contain 7.40% moisture, 30.10% crude protein, 3.60% crude fat, 6.90% crude fiber, ash 3.90% crude fiber and 48.10% nitrogen free extract [22]. Alfalfa is used as a forage crop and its seeds were consumed by humans [21]. Information on the supplementation of alfalfa seed flour in bakery products is insufficient; therefore, the objective of this research was to evaluate the effect of alfalfa seed flour supplementation on the quality characteristics of refined wheat flour–based biscuits.

## 2. Materials and Methods

### 2.1. Procurement of Raw Material

Refined wheat flour, alfalfa seeds and other ingredients used in biscuits preparation including sugar, shortening (vegetable ghee), skimmed milk powder, glucose, raising agents (sodium bicarbonate and ammonium bicarbonate), and emulsifying agents (lecithin and glycerol-mono-stearate) were obtained from local market.

### 2.2. Preparation of Alfalfa Seeds Flour (ASF)

Alfalfa seeds were cleaned and freed of broken seed, dust and other foreign materials manually and were washed with tap water. Clean alfalfa seeds were milled in laboratory mill with the size of 0.07 mm (Tecator Cyclotec sample mill Model No. 1093) and the flour was packed in thick polyethylene bags.

### 2.3. Preparation of Composite Flour Blends

Composite flour was prepared from refined wheat flour supplemented with alfalfa seeds flour at different levels and biscuits were prepared from supplemented flour with a little modification of the method described in [23] is indicated in Table 1.

### 2.4. Method

A known weight of vegetable ghee and powdered sugar was mixed together at medium speed to get light and soft cream. Then, the flour, skimmed milk powder, glucose, raising agents (sodium bicarbonate and ammonium bicarbonate) and emulsifying agents (lecithin and glycerol-mono-stearate) were added to the cream paste and mixed for obtaining uniform smooth dough. The dough was rolled out using a rolling pin. Biscuits were cut out into round shape of about 4.5 cm in diameter with a biscuit cutter. Baking was done at 175–185 °C for 10–12 min in baking oven. Biscuits were permitted to cool at ambient temperature for 8–10 min and were sealed in polyethylene bags for quality evaluation.

### 2.5. Physical, Chemical and Mineral Analysis

Physical characteristics of biscuits, such as diameter (width), thickness and spread factor were determined using the standard method described in [23]. Spread factor (*SF*) was calculated with the following formula. Where, *D* is the diameter, *T* denoted thickness and *CF* is a correction factor at constant atmospheric pressure and its value is 1.

*SF* = *D*/*T* × *CF* × 10 (1)

Chemical analysis of biscuits was determined according to the standard method of [23]. Difference method was used to calculate the nitrogen free extract [17].

Mineral contents were determined according to standard wet digestion method described in [24]. The mixture was cooled and filtered through filter paper and measured absorbance with the use of atomic absorption spectrophotometer (Model No. 2380, Perkin Elmer, Mercury Drive, Champaign, IL, USA). For potassium determination absorption flame photometer (Model PFP7, JENWAY, Nyavor K, Staffordshire, UK) was used.

### 2.6. Sensory Evaluation

A panel of 10 well-trained judges was selected to assess acceptability of biscuits. The biscuits were tested for different sensory quality characteristics such as, color, taste, texture and overall acceptability with the use of nine-point hedonic scales of [25].

### 2.7. Statistical Analysis

The experiment was carried out using a completely randomized design (CRD) with three replications with the using of software MSTAT-C for analysis of variance (ANOVA) and tested at the 5% significance level by [26].

## 3. Results and Discussion

### 3.1. Proximate Composition of Refined Wheat Flour and Alfalfa Seed Flour

The proximate composition of the refined wheat flour and alfalfa seed flour was determined as shown in Table 2. From the table, the results clearly exhibited that refined wheat flour is in close agreement with the findings of [27]. Similarly from Table 2, the alfalfa seed flour results obtained are in partial concurrence with the results of [22]. The little variation in the proximate composition might be due to a variety of differences and climatic conditions [28]. The data showed that alfalfa seed flour contained eminent contents of crude protein, crude fat, crude fiber and ash as compared to refined wheat flour, except or the nitrogen free extract and moisture content.

### 3.2. Physical Characteristics (Diameter, Thickness, and Spread Factor) of Control and Supplemented Biscuits

The average result of the diameter in the physical characteristics of control and supplemented biscuits is shown in the Table 3. The maximum mean value (47.25 mm) was recorded in M_S0_ whereas the minimum mean value (42.89 mm) was noted in M_S4_ (Table 3). Statistical analysis revealed that the diameter of biscuits had significantly (*p* < 0.05) decreased with the gradual increase in alfalfa seed flour supplementation. The decrease in the diameter may be attributed to an increased protein content in the flour blend as reported by [29]. These results are in accordance with the findings of [15]. Tiwari et al. [30] found that the diameter of biscuits decreased with the increase in the dehulled pigeon pea flour inclusion at different levels in wheat flour. Table 3 shows the average thickness results of control and supplemented biscuits. The maximum mean value (8.10 mm) was observed in M_S4_ whereas the minimum mean value (7.53 mm) was found in M_S0_ (Table 3).

Statistical analysis disclosed that the thickness of supplemented biscuits had significantly (*p* < 0.05) increased compared to that of control, except M_S1_ which contained 5% alfalfa seed flour supplementation. The results achieved are in line with the findings of [31]. Abu-Salem et al. [32] found that supplementation of wheat flour with Bambara groundnut flour increased the thickness of the biscuits. Similarly, the average results of the spreading factor in the physical characteristics of control and supplemented biscuits are also mentioned in Table 3. The maximum mean value (62.76) was noted in M_S0_ whereas the minimum mean value (53.12) was noted in M_S4_ (Table 3). Statistical analysis showed that the spread factor of biscuits had significantly (*p* < 0.05) decreased with the progressive increase in alfalfa seed flour supplementation. The decrease in the spread factor may be attributed to the effect of the composite flour which forms aggregates with an increased number of hydrophilic sites struggling for the limited free water in the biscuit dough [15]. Rapid partitioning of free water to hydrophilic sites throughout mixing increased dough viscosity and reduced the spread factor [33]. The results obtained were in complete agreement with the findings of [31,34] found that the spread factor of biscuits decreased with the increase in Bengal gram flour incorporation at different levels to the wheat flour.

### 3.3. Proximate Composition of Control and Supplemented Biscuits

The average results of the moisture content of control and supplemented biscuits are shown in Table 4. The maximum mean value (3.57%) was found in M_S0_ and the minimum mean value (3.26%) was noted in M_S4_ (Table 4). Statistical analysis disclosed that the moisture content of the supplemented biscuits had significantly (*p* < 0.05) decreased compared to that of the control biscuits, except M_S1_ which contained 5% alfalfa seed flour supplementation. The moisture content of the flour blend decreased with the addition of alfalfa seed flour, because the moisture content of the alfalfa flour is lower than that of the refined wheat flour. The present results are in agreement with the findings of [35]. Banureka et al. [36] found that the moisture content of biscuits increased with the increase in the soybean flour inclusion at different levels in the wheat flour. Similarly, for the crude protein content, the maximum mean value (15.30%) was recorded in M_S4_ while the minimum mean value (10.19%) was observed in M_S0_ (Table 4). Statistical analysis revealed that the crude protein content of the biscuits had significantly (*p* < 0.05) increased with the gradual increase in the alfalfa seed flour supplementation. The crude protein content increased in the flour blend due to the addition of the alfalfa seed flour, because of the higher crude protein content in the alfalfa seed flour as compared to the refined wheat flour. The present results are in complete agreement with the findings of [37]. Onoja et al. [38] reported that biscuits prepared from the blend of fermented legume and wheat flour had a higher protein content compared to biscuits prepared from 100% wheat flour.

For the crude fiber content, the average results of the control and supplemented biscuits are shown in Table 4. The maximum mean value (1.62%) was observed in M_S4_ whereas the minimum mean value (0.73%) was noted in M_S0_ (Table 4). Statistical analysis disclosed that the crude fiber content of biscuits had significantly (*p* < 0.05) increased with the progressive increase in alfalfa seed flour supplementation. The crude fiber content in the flour blend increased due to the addition of alfalfa seed flour, because the alfalfa seed flour has a higher crude fiber content than refined wheat flour. Comparable results were achieved by [39,40] found that biscuits prepared from the blend of mung bean and wheat flour had a higher crude fiber content compared to biscuits prepared from 100% wheat flour. Therefore, for the crude fat content, the maximum mean value (21.59%) was recorded in M_S4_ while the minimum mean value (17.46%) was found in M_S0_ (Table 4). Statistical analysis showed that the crude fat content of biscuits had significantly (*p* < 0.05) increased with the gradual increase in the alfalfa seed flour supplementation. The crude fat content of the flour blend is increased with the inclusion of alfalfa seed flour, because the crude fat content of alfalfa seed flour is high compared to refined wheat flour. These results are in accordance with the findings of [41,42] found that supplementation with high-oleic sunflower seed and hull-less barley flour increased the crude fat content of whole wheat flour-based biscuits.

Table 4 shows that the maximum mean value of the ash content of the control and supplemented biscuits (1.92%) is noted in M_S4_ while the minimum mean value (1.37%) is observed in M_S0_. Statistical analysis disclosed that the ash content of biscuits had significantly (*p* < 0.05) increased with the progressive increase in alfalfa seed flour supplementation. The increase in ash content could be attributed to the increased inclusion of alfalfa seed in the flour blend. This may be due to the fact that the ash content of alfalfa seed flour is high compared to refined wheat flour. The results obtained are in complete agreement with the findings of [43,44] indicated that the ash content of bread increased with the increase in soybean flour at different inclusion levels in the whole wheat flour. Similarly, for the nitrogen free extract, the maximum mean value (66.90%) was found in M_S0_ whereas the minimum mean value (56.32%) was noted in M_S4_ (Table 4). Statistical analysis disclosed that the nitrogen free extract of biscuits had significantly (*p* < 0.05) increased with the progressive increase in alfalfa seed flour supplementation. The increase in the nitrogen free extract could be due to increased incorporation of alfalfa seed in the flour blend. This may be due to the fact that the nitrogen free extract of alfalfa seed flour is low compared to refined wheat flour. These results were in agreement with the findings of [37,45] found that supplementation with soybean flour increased the nitrogen free extract of whole wheat flour–based bread.

### 3.4. Mineral Contents of Control and Supplemented Biscuits

The mineral content of wheat flour and alfalfa seed flour were checked and are shown in Table 5. The average maximum mean value of potassium (145.00 mg/100 g) is noted in M_S4_ while the minimum mean value (105.30 mg/100 g) is observed in M_S0_. Statistical analysis disclosed that the potassium content of biscuits had significantly (*p* < 0.05) increased with the increase in alfalfa seed flour supplementation. The increase in potassium content could be due to the increased inclusion of alfalfa seed in the flour blend. This may be due to the fact that the potassium content of alfalfa seed flour is high compared to refined wheat flour. The present results are in complete agreement with the findings of [39,40,41,42] found that supplementation with high-oleic sunflower seed and hull-less barley flour increased the potassium content of whole wheat flour-based biscuits.

Similarly, the average result of the magnesium content of the control and supplemented biscuits is shown in Table 5. The maximum mean value (26.64 mg/100 g) was recorded in M_S4_ while the minimum mean value (14.65 mg/100 g) was found in M_S0_ (Table 5). Statistical analysis showed that the magnesium content of the biscuits had significantly (*p* < 0.05) increased with the gradual increase in alfalfa seed flour supplementation. The increase in the magnesium content could be due to the increased inclusion of alfalfa seed in the flour blend. This may be due to the fact that the magnesium content of alfalfa seed flour is high compared to refined wheat flour. These results are in accordance with the findings of [38,39,40,41,42] found that supplementation with high-oleic sunflower seed and hull-less barley flour increased the magnesium content of whole wheat flour-based biscuits.

The maximum mean value of the calcium content (79.60 mg/100 g) was observed in M_S4_, while the minimum mean value (43.91 mg/100 g) was noted in M_S0_ (Table 5). Statistical analysis showed that the calcium content of biscuits had significantly (*p* < 0.05) increased with the progressive increase in alfalfa seed flour supplementation. The increase in the calcium content could be due to the increased incorporation of alfalfa seed in the flour blend. This may be due to the fact that the calcium content of alfalfa seed flour is high compared to refined wheat flour. Comparable results were achieved by [15,46] found that biscuits prepared from the blend of germinated fenugreek seed and wheat flour had a high protein content compared to biscuits prepared from 100% wheat flour.

However, for iron content, the maximum mean value (7.93 mg/100 g) was recorded in M_S4_ while the minimum mean value (3.74 mg/100 g) was observed in M_S0_ (Table 5). Statistical analysis revealed that the iron content of biscuits had significantly (*p* < 0.05) increased with the gradual increase in alfalfa seed flour supplementation. The increase in iron content could be due to the increased inclusion of alfalfa seed in the flour blend. This may be due to the fact that the iron content of alfalfa seed flour is high compared to refined wheat flour. Similarly, the maximum mean value of the zinc content (1.60 mg/100 g) was found in M_S4_ while the minimum mean value (0.94 mg/100 g) was noted in M_S0_ (Table 5). Statistical analysis showed that the zinc content of biscuits had significantly (*p* < 0.05) increased with the progressive increase in alfalfa seed flour supplementation. The increase in the zinc content could be due to the increased inclusion of alfalfa seed in the flour blend. This may be due to the fact that the zinc content of alfalfa seed flour is high compared to refined wheat flour. These results are in agreement with the findings of [43,44,45,46] found that substitution with germinated fenugreek seed flour increased zinc content of wheat flour–based biscuits.

### 3.5. Sensory Evaluation of Control and Supplemented Biscuits

**Figure 1:**
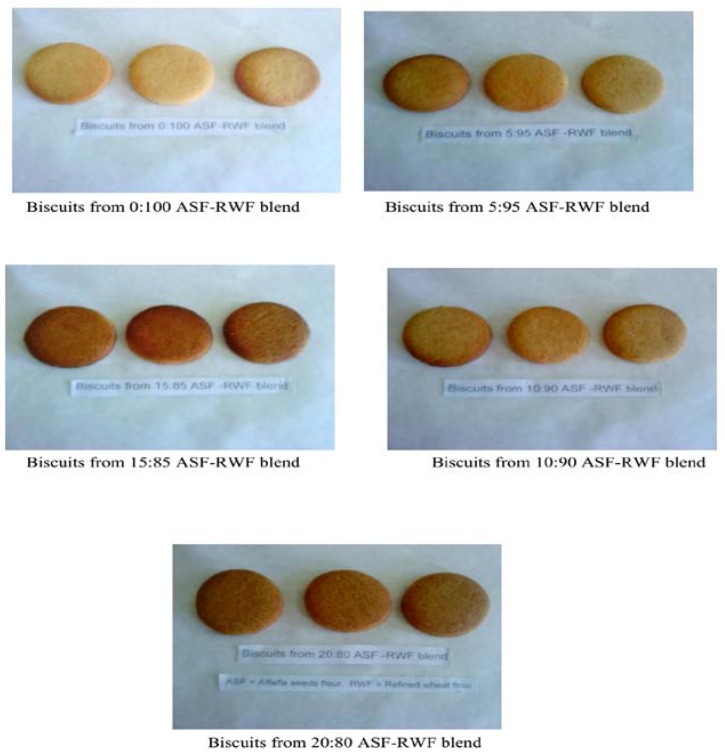
Control and alfalfa seed flour–supplemented biscuits.

Figure 1 shows the color of the control and supplemented biscuits. The average results for the color of the control and supplemented biscuits are shown in Table 6. The maximum mean value (8.00) was observed in M_S0_ while the minimum mean value (5.60) was recorded in M_S4_. Statistical analysis showed that the supplementation had significantly (*p* < 0.05) influenced color of the biscuits. The dark brown color dominated with the gradual increase in alfalfa seed flour. This may be due to the reaction between reducing the sugar and amino acids (Maillard reaction) and caramelization as reported by [47]. These results are in accordance with the findings of [32,45] reported a dark brown color in whole wheat flour-based biscuits supplemented with soybean flour. Similarly, the maximum mean value for taste (8.25) was noted in M_S0_ whereas the minimum mean value (5.00) was found in M_S4_ (Table 6). Statistical analysis disclosed that the taste score of biscuits varied significantly (*p* < 0.05) between the treatments. Results exhibited a decrease in the quality of alfalfa seed flour–supplemented biscuits in terms of taste. This decrease may be attributed to the beany flavor of alfalfa seed flour. Beany flavor is usually linked with legumes [48]. In legumes, hydro-peroxides such as aldehydes, ketones and alcohols are produced from linoleic and linolenic acid through enzymatic breakdown by lipoxygnases which may be responsible for the beany flavor [49,50]. The present results are in close agreement with the findings of [34,46,47,48,49,50] found that the quality of biscuits in terms of taste decreased with the increase in soybean and rice bran flour supplementation.

The average results for the texture of the control and supplemented biscuits are shown in Table 6. The maximum mean value (7.85) was recorded in M_S0_ whereas the minimum mean value (5.38) was noted in M_S4_ (Table 6). Statistical analysis revealed that the texture score of the biscuits had significantly (*p* < 0.05) decreased with the progressive increase in alfalfa seed flour supplementation. This decrease may be due to the increased crude fiber content of alfalfa seed flour in the flour blend. The results obtained are in accordance with the findings of [31,32] found that the quality of biscuits in terms of texture decreased with the increased incorporation of bambara groundnut flour.

Therefore, the maximum mean value of the overall acceptability (8.10) was found in M_S0_ while the minimum mean value (5.32) was observed in M_S4_ (Table 6). Statistical analysis showed that the overall acceptability quality score of biscuits varied significantly (*p* < 0.05) between the treatments. Results revealed a decreased quality of alfalfa seed flour–supplemented biscuits in terms of overall acceptability. Results obtained are in line with the findings of [30,32] also reported similar results when they studied dehulled pigeon pea flour–supplemented biscuits. Even though biscuits prepared from composite flour (supplemented biscuits) were of inferior quality in terms of sensory attributes, when assigned for comparison with those prepared from refined wheat flour (control biscuits), the score given by the trained panel of judges remained at fairly good level for all parameters, showing acceptability of the supplemented biscuits.

## 4. Conclusions

The present research work confirmed that the recommended supplementation of refined wheat flour should be up to a maximum of 10%, which could produce acceptable biscuits with an appropriate nutritional profile. The physical characteristics of alfalfa seed flour–supplemented biscuits portrayed variable results. The thickness of the biscuits increased while the width and spread factor decreased. Supplementation of alfalfa seed flour significantly influenced the proximate composition of refined wheat flour–based biscuits. Crude protein, crude fiber, crude fat and ash contents increased whereas moisture content and nitrogen free extract decreased. Mineral contents (potassium, magnesium, calcium, iron and zinc) of biscuits increased with the increase in alfalfa seed flour supplementation. Sensory evaluation of the biscuits showed that the quality score in terms of color, taste, texture and overall acceptability decreased with the increased supplementation of alfalfa seed flour.

## Figures and Tables

**Table 1:** Weight of ingredients used in biscuits preparation.

Ingredients	Weight (g)
Flour	500
Sugar	150
Shortening	125
Skimmed milk powder	25
Glucose	15
Salt	3
Sodium bicarbonate	2
Ammonium bicarbonate	5
Lecithin	2
Glycerol-mono-sterate	3

**Table 2:** Proximate composition of refined wheat flour (RWF) and alfalfa seed flour (ASF).

Constituents	RWF	ASF
**Moisture %**	12.43 ± 0.18	5.79 ± 0.13
**Crude protein %**	11.52 ± 0.08	29.49 ± 0.19
**Crude fat %**	1.61 ± 0.06	12.71 ± 0.15
**Crude fiber %**	0.71 ± 0.02	5.53 ± 0.12
**Ash %**	1.43 ± 0.05	4.80 ± 0.07
**Nitrogen free extract %**	70.83 ± 0.13	41.73 ± 0.216

Note: Values are means of three replications ± standard deviation.

**Table 3:** Physical characteristics of control and supplemented biscuits.

Treatments	Diameter (mm)	Thickness (mm)	Spread Factor
**M_S0_** (100% RWF + 0% ASF)	47.25 ^a^ ± 0.24	7.53 ^c^ ± 0.27	62.76 ^a^ ± 0.21
**M_S1_** (95% RWF + 5% ASF)	45.18 ^b^ ± 0.19	7.66 ^bc^ ± 0.10	58.98 ^b^ ± 0.15
**M_S2_** (90% RWF + 10% ASF)	44.35 ^bc^ ± 0.41	7.78 ^b^ ± 0.29	57.10 ^bc^ ± 0.33
**M_S3_** (85% RWF + 15% ASF)	43.86 ^cd^ ± 0.32	7.86 ^ab^ ± 0.11	55.85 ^c^ ± 0.18
**M_S4_** (80% RWF + 20% ASF)	42.89 ^d^ ± 0.20	8.10 ^a^ ± 0.17	53.12 ^d^ ± 0.12
**C.V (%)**	2.51	2.19	3.15

RWF, refined wheat flour; ASF, alfalfa seed flour. Values are means of three replications ± standard deviation and significantly different at (*p* < 0.05).

**Table 4:** Proximate composition of control and supplemented biscuits.

Treatments	Moisture %	C. Protein %	C. Fiber %	C. Fat %	Ash %	N F E %
**M_S0_** (100% RWF + 0% ASF)	3.57 ^a^ ± 0.09	10.19 ^e^ ± 0.38	0.73 ^d^ ± 0.11	17.46 ^d^ ± 0.17	1.37 ^d^ ± 0.13	66.90 ^a^ ± 0.19
**M_S1_** (95% RWF + 5% ASF)	3.49 ^a^ ± 0.11	12.02 ^d^ ± 0.16	1.17 ^c^ ± 0.16	18.88 ^c^ ± 0.22	1.54 ^c^ ± 0.10	62.89 ^b^ ± 0.42
**M_S2_** (90% RWF + 10% ASF)	3.39 ^b^ ± 0.22	13.13 ^c^ ± 0.41	1.36 ^b^ ± 0.09	19.53 ^c^ ± 0.16	1.66 ^bc^ ± 0.05	60.94 ^c^ ± 0.57
**M_S3_** (85% RWF + 15% ASF)	3.31 ^c^ ± 0.10	14.19 ^b^ ± 0.27	1.52 ^ab^ ± 0.11	20.47 ^b^ ± 0.27	1.78 ^b^ ± 0.11	58.73 ^d^ ± 0.51
**M_S4_** (80% RWF + 20% ASF)	3.26 ^c^ ± 0.14	15.30 ^a^ ± 0.19	1.62 ^a^ ± 0.07	21.59 ^a^ ± 0.34	1.92 ^a^ ± 0.08	56.32 ^e^ ± 0.34
**C.V (%)**	3.81	4.27	5.23	4.85	3.46	2.91

RWF: refined wheat flour; ASF: alfalfa seed flour. Values are means of three replications ± standard deviation and significantly different at (*p* < 0.05).

**Table 5:** Mineral contents of control and supplemented biscuits.

Treatments	K (mg/100 g)	Mg (mg/100 g)	Ca (mg/100 g)	Fe (mg/100 g)	Zn (mg/100 g)
**M_S0_** (100% RWF+ 0% ASF)	105.30 ^e^ ± 0.81	14.65 ^d^ ± 0.26	43.91 ^d^ ± 1.13	3.74 ^e^ ± 0.13	0.94 ^d^ ± 0.09
**M_S1_** (95% RWF + 5%ASF)	118.50 ^d^ ± 0.93	19.80 ^c^ ± 0.13	60.14 ^c^ ± 0.82	5.30 ^d^ ± 0.21	1.29 ^c^ ± 0.11
**M_S2_** (90% RWF + 10%ASF)	127.20 ^c^ ± 0.49	22.49 ^b^ ± 0.31	64.98 ^c^ ± 1.29	6.24 ^c^ ± 0.10	1.36 ^bc^ ± 0.17
**M_S3_** (85% RWF + 15%ASF)	135.40 ^b^ ± 1.11	24.33 ^b^ ± 0.17	71.53 ^b^ ± 0.54	7.14 ^b^ ± 0.32	1.43 ^b^ ± 0.13
**M_S4_** (80% RWF + 20%ASF)	145.00 ^a^ ± 0.74	26.64 ^a^ ± 0.22	79.60 ^a^ ± 0.79	7.93 ^a^ ± 0.09	1.60 ^a^ ± 0.10
**C.V (%)**	1.84	0.72	2.32	1.59	1.67
**Wheat seed flour**	486 ± 0.12	166 ± 0.18	40.8 ± 0.15	4.7 ± 0.25	3.5 ± 0.13
**Alfalfa seed flour**	26.1 ± 0.09	8.9 ± 0.13	10.6 ± 0.11	0.3 ± 0.10	0.3 ± 0.01

RWF, refined wheat flour; ASF, alfalfa seed flour. Values are means of three replications ± standard deviation and significantly different at (*p* < 0.05).

**Table 6:** Sensory evaluation of control and supplemented biscuits.

Treatments	Color	Taste	Texture	O. Acceptability
**M_S0_** (100% RWF + 0% ASF)	8.00 ^a^ ± 0.73	8.25 ^a^ ± 0.36	7.85 ^a^ ± 0.47	8.10 ^a^ ± 0.47
**M_S1_** (95% RWF + 5%ASF)	7.80 ^a^ ± 0.15	7.90 ^a^ ± 0.52	7.13 ^ab^ ± 0.62	7.61 ^ab^ ± 0.38
**M_S2_** (90% RWF + 10%ASF)	7.29 ^ab^ ± 0.33	7.74 ^a^ ± 0.52	6.56 ^b^ ± 0.56	7.19 ^b^ ± 0.54
**M_S3_** (85% RWF + 15%ASF)	6.68 ^b^ ± 0.26	6.24 ^b^ ± 0.23	5.48 ^c^ ± 0.58	6.13 ^c^ ± 0.63
**M_S4_** (80% RWF + 20%ASF)	5.60 ^c^ ± 0.39	5.00 ^c^ ± 0.49	5.38 ^c^ ± 0.47	5.32 ^c^ ± 0.30
**C.V (%)**	7.13	8.74	9.79	7.92

RWF, refined wheat flour; ASF, alfalfa seed flour. Values are means of three replications ± standard deviation and significantly different at (*p* < 0.05).
